# Effectiveness of intravenous lidocaine versus intravenous morphine for patients with renal colic in the emergency department

**DOI:** 10.1186/1471-2490-12-13

**Published:** 2012-05-04

**Authors:** Hassan Soleimanpour, Kamaleddin Hassanzadeh, Hassan Vaezi, Samad EJ Golzari, Robab Mehdizadeh Esfanjani, Maryam Soleimanpour

**Affiliations:** 1 Emergency Medicine Department, Tabriz University of Medical Sciences, Daneshgah Street, Tabriz -51664, IR IRAN; 2 Urology Department, Tabriz University of Medical Sciences, Daneshgah Street, Tabriz -51664, IR IRAN; 3 Medical Philosophy and History Research Center, Tabriz University of Medical Sciences, Daneshgah Street, Tabriz -51664, IR IRAN; 4 Students’ Research Committee, Tabriz University of Medical Sciences, Daneshgah Street, Tabriz -51664, IR IRAN; 5 Neurosciences Research Center, Tabriz University of Medical Sciences, Daneshgah Street, Tabriz -51664, IR IRAN; 6 Gastroenterology Research Center, Tabriz University of Medical Sciences, Daneshgah Street, Tabriz -51664, IR IRAN

**Keywords:** Visual Analogue Pain Scale (VAS), Renal colic, Lidocaine, Morphine

## Abstract

**Background:**

Despite the fact that numerous medications have been introduced to treat renal colic, none has been proven to relieve the pain rapidly and thoroughly. In this study, we aimed at comparing the effects of intravenous lidocaine versus intravenous morphine in patients suffering from renal colic.

**Methods:**

In a prospective randomized double-blind clinical trial performed in the emergency department of Imam Reza educational hospital of Tabriz, Iran, we studied 240 patients, 18–65 years old, who were referred due to renal colic. Patients were divided into two groups. In group I (120 people) single-dose intravenous lidocaine (1.5 mg/kg) was administered and in group II (120 people) single-dose intravenous morphine (0.1 mg/kg) was administered slowly. Visual Analogue Pain Scale (VAS) was recorded while admission, 5, 10, 15 and 30 minutes after injection. Statistical data and results were studied using descriptive statistics as percentage and Mean ± SD. To compare the response to treatment, Mann–Whitney *U*-test was used in two groups. Consequently, the data were analyzed using the SPSS16 software.

**Results:**

Pain score measured in two groups five minutes after the injection of lidocaine and morphine were 65 % and 53 % respectively (95% CI 0.60 - 0.69, CI 0.48 – 0.57, p = 0.0002).108 (90 %) patients (95 % CI 0.84 – 0.95) from group I and 84 (70%) patients (95 % CI 0.62 - 0.78) from group II responded appropriately at the end of the complete treatment. The difference was statistically significant (p = 0.0001).

**Conclusions:**

Changing the smooth muscle tone and reducing the transmission of afferent sensory pathways, lidocaine causes a significant reduction in pain.

**Trial registration:**

Clinical Trials IRCT138901042496N3

## Background

Affecting 1-5 % of the population in industrialized countries, renal colic is considered as a major concern in medicine. Renal colic has been reported to be experienced by 20 % of white males and 5-10 % of white females [1]. The classic presentation of acute renal colic includes sudden pain onset radiating from the flank to the lower extremities which is usually accompanied by microscopic hematuria (85 % of cases), nausea and vomiting . Costovertebral angle tenderness is a common finding as well [2].

To relieve the pain until being discharged or undergoing the required operation is mostly performed in emergency departments [1]. To achieve this, numerous medications including antiemetics, narcotics, non-steroidal anti-inflammatory drugs, antispasmodics, anti-diuretics, ketorolac, nifedipine, prednisone, acetaminophen and prochlorperazine have been introduces [2].

Lidocaine, being an appropriate choice in treating visceral and central pain, might also be useful wherever narcotics are inefficient or lead to undesirable side effects. Intravenous lidocaine is effective in controlling neuropathic pains such as: diabetic neuropathy, post-operative pain, post-herpetic pain, headaches and neurological malignancies [3,4]. Therefore we aimed at investigating and reviewing the analgesic effects of intravenous lidocaine compared with intravenous morphine in patients with renal colic.

## Methods

To perform a prospective randomized double-blind clinical trial we evaluated the sample size of 100 people for each group. This study was approved by Ethics Committee of “Tabriz University of Medical Sciences”. The trial was registered in “IRCT”. Additionally, all aspects of the present study plan were explained to patients and written informed consents were obtained consequently.

Considering pain incidence ratios of 29.3 % and 12 % for the control and intervention groups respectively, confidence interval of 0.05, power of 20 % and loss probability of 20 % throughout the follow-up period using the following formula, a sample size of 100 people were calculated

(1) $$\;\text{N}=\text{ 2 }\left(\text{z }1-\alpha /2\;*\text{ z }\beta \right)\text{ 2 }*\left\{\text{P }*\left(1-\text{ P}\right)\right\}/\left(\text{p1 }–\text{ p}2\right)2$$

(2) $$\text{P }=\left(\text{p1 }+\text{ p}2\right)/2$$

(3) N = 86 + 15 ≠100

As the number of the patients with renal colic referring to the emergency department of Imam Reza Hospital is abundant [5], the sample size was considered as 120 people for both groups and a total number of 240.

Using the website of http://www.randomization.com 240 letters of A and B were evenly (120) produced and patients were allocated to one of the two groups of A (Group I) and B (Group II), in the order provided by the site.

Patients were evenly divided into two groups of I, receiving intravenous lidocaine (n = 120), and II, receiving intravenous morphine (n = 120). As previously mentioned the study was double-blind and the executive of the research project was unaware of the obtained figures using online software from http://www.randomization.com. Firstly, patients entering emergency department with complaint of flank pain were identified, interviewed and examined. Later, patients with pain radiating to genitalia and groin, nausea, vomiting, urinary irritation symptoms and tenderness in costovertebral angle were selected.

To provide the patients with required analgesia and to avoid any inconvenience regarding pain management, initial diagnosis was made based on clinical findings (unilateral abdominal pain radiating to the genitalia) associated with a positive urine analysis for hematuria. However diagnosis was later confirmed using Kidney, Ureter, and Bladder (KUB) radiographies and sonography studies. Since all patients experienced unilateral abdominal pain and their urine analyses were positive for hematuria, no further diagnostic criteria for renal colic were set throughout the study [1].

All patients aged 18 to 65 years, and did not have history of renal, hepatic or cardiac disease (Ischemic Heart Disease).

Patients with history of allergy to lidocaine and morphine, an inability or unwillingness to provide written informed consent and pregnant females were excluded from the study as well. Patients whose pain did not relieve using lidocaine or morphine were administered supplementary drugs. Then, method of drug administration, the reason for prescription and possible complications were explained to the patients and it was emphasized that using either lidocaine or morphine is safe.

After explaining method of treatment and obtaining informed written consent, 12 lead electrocardiograms were taken from all patients to make sure no history of heart diseases existed. Later, patients were monitored by an emergency medicine specialist throughout the procedure. Additionally patients were asked to identify the degree of their pain using Visual Analogue Pain Scale (VAS), zero represented painless and 10 was the worst experienced pain. Vital signs and other symptoms of patients including nausea, vomiting, dysuria, hematuria, and etc. were recorded. Firstly, all the enrolled patients received intravenous metoclopramide (0.15 mg/kg). Later, to reduce pain in patients suffering from renal colic in group I lidocaine solution (1.5 mg/kg) and in group II morphine solution (0.1 mg/kg) were administered intravenously slowly (Each syringe contained 10 mL of solution, either 10 mg of morphine or 200 mg of lidocaine %2 (1 mL = 20 mg)). The trial was considered accomplished when either the patient had a pain score of less than 3 for 30 minutes after the last analgesic dose or the 10 mL of solution in the syringe was used up.

As fluid therapy is not commonly used in our center to treat patients with renal colic and it is restored only for patients who are dehydrated or have sepsis, our patients received very little amount of fluid, (10 mL) administered only by injection [6].

### Notice

All the processes of injection and filling in the questionnaires were performed by another person who was a specialist (not involved in research project) and blinded to the injected drug as well as the patients groupings. Patients were blinded to the injected medications as well. The doses required for the administered medication were calculated by another colleague. Hence; neither the patients nor the administrator were aware of the medications used.

Consequently, 5, 10, 15 and 30 minutes after injection, incidence of pain in patients was recorded based on Visual Analog Scale (VAS). Statistical data and results were expressed using descriptive statistics as percentage and Mean ± SD. To compare the response to treatment, statistical Mann–Whitney *U*-test was performed in two groups. Kolmogroff Smirnov test was used for normality of variables and QQ plot test was used in cases of necessity. The data were analyzed using the SPSS16 software.

## Results

Eighty six patients of studied samples were male and thirty four were female in group I. In group II, ninety patients were male and thirty were female. There was no statistically significant difference between them regarding gender distribution (p = 0.559) (Table 1).

**Table 1:** Demographics characteristics of both groups

		**Group I**	**Group II**	***P*-value**
Sex	Male	71.7 %	28.3 %	0.559
	Female	75 %	25 %	
age		35.23 ± 12.37	37.71 ± 11.08	0.104
Hydronephrosis	left kidney	52 (43.3 %)	52 (43.3 %)	0.684
	right kidney	57 (47.5 %)	53 (44.2 %)	
	did not have hydronephrosis	11 (9.2 %)	15 (12.5 %)	
history of kidney stone and renal colic	Recurrent Stone	49 (41.2 %)	64 (53.3 %)	0.06
	First Stone	70 (58.8 %)	56 (46.7 %)	
Stone side	right kidney	60 (50 %)	57 (47.5 %)	0.698
	left kidney	60 (50 %)	63 (52.5 %)	

Mean ages of the patients were 35.23 ± 12.37 years and 37.71 ± 11.08 years in group I and II respectively. The differences was not statistically significant (p = 0.104) (Table 1).

There was no statistically significant difference between mean patient pain scores before drug administration of two groups (p = 0.365). Pain relief in lidocaine group was statistically significant compared to the morphine group (p = 0.0001) (Table 2).

**Table 2:** Comparison of the mean value of pain reduction between two groups

	**Group I**	**Group II**	***P*-value**
primary VAS	9.65 ± 0.88	9.74 ± 0.63	0.365
VAS^5^	3.18 ± 2.27	4.45 ± 2.16	0.0001
VAS^10^	1.83 ± 1.59	2.89 ± 2.07	0.0001
VAS^15^	1.37 ± 1.32	2.55 ± 1.52	0.0001
VAS^30^	1.13 ± 1.15	2.23 ± 1.57	0.0001

108 patients (90 %) in group I responded to lidocaine successfully whereas in group II, 84 patients (70 %) responded properly to morphine, at the end of the study it was demonstrated that there were more considerable respond and pain relief in lidocaine group which was statistically significant (p = 0.0001).

In the present study, patients were also evaluated regarding having history of kidney stone, renal colic as (with a history of referring to the hospital or recurrent stone, and first referral-first stone), side effects (all recorded side effects in both groups were mild and temporary), stone side and hydronephrosis (Tables 1,3).

**Table 3:** Percentage of patients reporting side effects

Group I	perioral numbness	3 (2.5 %)
	transient dizziness	10 (8.3 %)
	dysrathria	2 (1.7 %)
	Without side effect	105 (87.5 %)
Group II	hypotension	3 (2.5 %)
	vertigo	2 (1.7 %)
	nausea	9 (7.5 %)
	vomiting	2 (1.6 %)
	Without side effect	104 (86.7 %)

## Discussion

Since renal colic pain caused by urinary stones is severe and sudden, an appropriate treatment is of great necessity. Narcotics have been considered as main treatment of renal colic for years; they relieve pain with central effects on narcotic receptors. Although narcotics have adequate effects, there is possibility of their being misused, excessive drowsiness, and other central nervous system and gastrointestinal complications [7].

Recently, the benefits of non-steroidal anti-inflammatory drugs (NSAIDS) in renal colic have been reported suggesting them to be more effective compared to narcotic analgesics [8,9].

Renal colic pain is caused by endopelvic pressure and ureteral obstruction. Increase in renal blood flow following obstruction increases urine and endopelvic and ureter pressure. Prostaglandins cause increase in renal blood follow and smooth muscle spasm [10].

Nonsteroidal anti-inflammatory drugs, inhibiting cyclooxygenase enzyme, relieve colic pain. Their pain relief mechanism however differentiates them from narcotic analgesics. NSAIDs can relieve pain locally and centrally inhibiting prostaglandin synthesis [11].

In spite of the beneficial effects of nonsteroidal anti-inflammatory drugs on pain, NSAIDs can reduce renal blood flow and ureter pressure in acute ureteral obstruction [12].

Therefore, because in our country (Iran), intravenous nonsteroidal anti-inflammatory drugs are not available, we thought of an alternative intravenous drug, i.e. lidocaine, whose analgesic effects on various pains (postoperative, cancer and etc.) have been proven [3].

Being an amino amide local anesthetic, Lidocaine blocks the voltage dependant sodium channels and impulses in axons [13]. Lidocaine is a relatively safe medication, if used in low doses. Allergy to this drug however would increase the risk of cardiac arrhythmia and dyspnea in rare cases. Most side effects are related to its cumulative effects including: perioral numbness, dizziness, confusion, feeling hangover, and impaired speech [3].

Lidocaine is an effective and cheap drug with few side effects including dizziness, nausea and constipation. Prevalence of these complications however is less with lidocaine compared to narcotics and other analgesics [14].

On the other hand, side effects of lidocaine are predictable with a wide range of confidence. Due to the low half life, toxicity symptoms of lidocaine are transient and rapidly reversible. Based on the results of Rebecca Ferrini’s study on 100 patients, in most patients having received narcotics infusion of intravenous lidocaine was effective when the pain was central or visceral, and even when narcotics were not effective or accompanied unwanted and unacceptable complications, intravenous lidocaine could be useful [3].

In the study of Nicky Forov et al. on a pregnant woman with resistant renal colic, subcutaneous paravertebral nerve block was performed using lidocaine, in this study patient’s pain relieved considerably [15]. In our other case series study, from 8 patients suffering from renal colic resistant to treatment (morphine and NSAIDS), intravenous lidocaine relieved the pain in 7 patients significantly with no considerable complications [6].

Other case studies have also been reported regarding using lidocaine in relieving pain in cancerous and renal colic patients. In a similar study carried out by Rebecca Ferrini et al. A single dose of lidocain was administered for reducing pain resistant to morphine in a patient suffering from primary neuroectodermal tumor. The patient had received other complementary medicines, in addition to morphine, such as: gabapentin, baclofen, amitriptyline, clonidine and clonazepam. Patient’s pain however considerably relieved after using a single dose of lidocaine (1.5 mg/kg) [3].

The only study which can be compared with ours regarding considerable pain reduction in short time is a study carried out by Mansuri Igochi et al. in which local injection of lidocaine in the most painful point was used in patients suffering from renal colic (control group received scopolamine). Considering response to treatment, lidocaine was more effective than scopolamine [12].

Fortunately, in our study no serious or life threatening complications were observed in patients of lidocaine group, therefore our study is similar to the study of Rebecca Ferrini et al.

Lidocaine causes change in sympathetic smooth muscle tone through reducing the transmission of afferent sensory pathways. Intravenous lidocaine causes considerable reduction in pain and can be a suitable alternative for cases in which narcotics are ineffective or associated with undesirable complications [16].

## Conclusion

Our study is unique considering the fact that it is the first clinical trial study being carried out on using intravenous lidocaine in patients suffering from renal colic.

The results of our study revealed that although morphine is used in patients suffering from renal colic as a drug of choice, due to the rapid response of patients to lidocaine (considering time and reduction in pain intensity compared to morphine) it can be of great importance in patients with renal colic.

On the other hand, we should note that the complete response rate to lidocaine treatment was significantly higher compared to morphine which could be considered as a reason for lidocaine to be used as a drug of choice with high priority in patients with renal colic.

## Abbreviations

VAS: Visual Analogue Pain Scale; NSAIDs: Non-steroidal anti-inflammatory drugs.

## Competing interests

The authors declare that they have no competing interests.

## Authors’ contributions

HS, KH and HV collected clinical data, reviewed the literature on the topic, and drafted the manuscript. SEJG, MS and RME participated in the design of the study and performed the statistical analysis. All of the authors were involved in patient management or the writing of the manuscript. All authors read and approved the final manuscript.

## Authors’ information

HS is Associate professor of Anesthesiology and Critical Care, Fellowship in Trauma Critical Care and CPR at the Department of Emergency Medicine, Tabriz University of Medical Sciences, Tabriz, Iran. He is also editorial board member of Emergency medicine journal (EGM) and Pakistan Journal of Biological Sciences (PJBS). KH is Associate professor of Urology at the Department of Urology, Tabriz University of Medical Sciences, Tabriz, Iran. HV and SEJG are residents of Emergency Medicine and Anesthesiology at the Department of Emergency Medicine and Anesthesiology depapartment, Tabriz University of Medical Sciences, Tabriz, Iran, respectively. RME is member of Neurosciences Research Center, Tabriz University of Medical Sciences, Tabriz, Iran. MS is member of Gastroenterology Research Centre, Tabriz University of Medical Sciences, Tabriz, Iran.

## Pre-publication history

The pre-publication history for this paper can be accessed here:

http://www.biomedcentral.com/1471-2490/12/13/prepub
